# Present and Future of Allogeneic Natural Killer Cell Therapy

**DOI:** 10.3389/fimmu.2015.00286

**Published:** 2015-06-03

**Authors:** Okjae Lim, Mi Young Jung, Yu Kyeong Hwang, Eui-Cheol Shin

**Affiliations:** ^1^Virology and Immunology Team, MOGAM Biotechnology Institute, Yongin, South Korea; ^2^Cell Therapy Center, GreenCross LabCell, Yongin, South Korea; ^3^Laboratory of Immunology and Infectious Diseases, Graduate School of Medical Science and Engineering, KAIST, Daejeon, South Korea

**Keywords:** natural killer cells, allogeneic, cancer immunotherapy, adoptive cell therapy, non-HLA-related donor

## Abstract

Natural killer (NK) cells are innate lymphocytes that are capable of eliminating tumor cells and are therefore used for cancer therapy. Although many early investigators used autologous NK cells, including lymphokine-activated killer cells, the clinical efficacies were not satisfactory. Meanwhile, human leukocyte antigen (HLA)-haploidentical hematopoietic stem cell transplantation revealed the antitumor effect of allogeneic NK cells, and HLA-haploidentical, killer cell immunoglobulin-like receptor ligand-mismatched allogeneic NK cells are currently used for many protocols requiring NK cells. Moreover, allogeneic NK cells from non-HLA-related healthy donors have been recently used in cancer therapy. The use of allogeneic NK cells from non-HLA-related healthy donors allows the selection of donor NK cells with higher flexibility and to prepare expanded, cryopreserved NK cells for instant administration without delay for *ex vivo* expansion. In cancer therapy with allogeneic NK cells, optimal matching of donors and recipients is important to maximize the efficacy of the therapy. In this review, we summarize the present state of allogeneic NK cell therapy and its future directions.

## Introduction

Cancer is a major threat for humans worldwide, with approximately 14 million new cases and 8.2 million cancer-related deaths in 2012 (1). Although most common cancer treatments include surgery, chemotherapy, and radiotherapy, unsatisfactory cure rates require new therapeutic approaches, especially for refractory cancers. For this purpose, cancer immunotherapies with various cytokines, antibodies, and immune cells have been clinically applied to patients to encourage their own immune system to help fight the cancer (2).

Adoptive cellular immunotherapies have employed several types of immune cells, including dendritic cells (DCs), cytotoxic T lymphocytes (CTLs), lymphokine-activated killer (LAK) cells, cytokine-induced killer (CIK) cells, and natural killer (NK) cells. Although there has been recent progress in DC therapy and CTL therapy, clinical applications are somewhat limited because cancer antigens must first be characterized and autologous cells must be used. By contrast, LAK cells, CIK cells, and NK cells have antigen-independent cytolytic activity against tumor cells. In particular, NK cells can be used from not only autologous sources but also allogeneic sources and, recently, allogeneic NK cells have been employed more often in cancer treatment. Whereas autologous NK cells from cancer patients may have functional defects (3), allogeneic NK cells from healthy donors have normal function and can be safely administered to cancer patients (4). Allogeneic NK cell therapy is particularly beneficial because it can enhance the anti-cancer efficacy of NK cells via donor–recipient incompatibility in terms of killer cell immunoglobulin-like receptors (KIRs) on donor NK cells and major histocompatibility complex (MHC) class I on recipient tissues.

## Biology of NK Cells and Their Receptors

Natural killer cells are innate lymphocytes that provide a first line of defense against viral infections and cancer (5). Human NK cells are recognized as CD3^−^CD56^+^ lymphocytes. They can be further subdivided into two subsets based on the surface expression level of CD56. The CD56^dim^ population with low-density expression of CD56 comprises approximately 90% of human blood NK cells and has a potent cytotoxic function, whereas the CD56^bright^ population (approximately 10% of blood NK cells) with high-density expression of CD56 displays a potent cytokine producing capacity and has immunoregulatory functions (6). The CD56^dim^ NK cell subset also expresses high levels of the Fc receptor for IgG (FcγRIII, CD16), which allows them to mediate antibody-dependent cellular cytotoxicity (ADCC) (7). NK cells comprise 5–15% of circulating lymphocytes and are also found in peripheral tissues, including the liver, peritoneal cavity, and placenta. Activated NK cells are capable of extravasation and infiltration into tissues that contain pathogens or malignant cells while resting NK cells circulate in the blood (8).

The NK cell activity is regulated by signals from activating and inhibitory receptors (9, 10). The activating signal is mediated by several NK receptors including NKG2D and natural cytotoxicity receptors (NCRs) (9–11). By contrast, NK cell activity is suppressed by inhibitory receptors, including KIRs, which bind to human leukocyte antigen (HLA) class I molecules on target cells (9, 10, 12). NKG2A is also an important inhibitory receptor binding to non-classical HLA molecule, HLA-E (13). If target cells lose or downregulate HLA expression (14), the NK inhibitory signal is abrogated, allowing NK cells to become activated and kill malignant targets. However, NK cell function is impaired in cancer patients by various mechanisms, particularly in tumor microenvironment (15).

Although NK cell activity is determined by the summation of signals from activating and inhibitory receptors, the inhibitory signal through KIRs is a main regulator of NK cell function particularly in allogeneic settings. Inhibitory KIRs have long cytoplasmic tails containing two immunoreceptor tyrosine-based inhibition motifs (ITIMs). Each KIR has its cognate ligand and consists of two (KIR2DL) or three (KIR3DL) extracellular Ig-domains. KIR2DL1 and KIR2DL2/3 recognize group 2 HLA-C (called C2, Lys80) and group 1 HLA-C (called C1, Asn80), respectively. KIR3DL1 recognizes HLA-Bw4 (16). The KIR repertoire on human NK cells is randomly determined and independent of the number and allotype of HLA class I ligands (17).

## Therapeutic Efficacy of Allogeneic NK Cells

### Role of allogeneic NK cells in hematopoietic stem cell transplantation

The antitumor activity of allogeneic NK cells has been demonstrated in the setting of hematopoietic stem cell transplantation (HSCT). Allogeneic HSCT is an established curative treatment for hematologic malignancies. In allogeneic HSCT, donor T cells contribute to graft-versus-host disease (GVHD) and graft-versus-tumor (GVT) effects (18). In T cell-depleted HSCT, however, donor NK cells are the major effector cells responsible for controlling residual cancer cells before T cell reconstitution (19, 20).

Natural killer cells are the first lymphoid population to recover after allogeneic HSCT. In the first month of transplantation, reconstituted NK cells represent the predominant lymphoid cells and play a crucial role in controlling the host immune system. Allogeneic NK cells prevent viral infections and restrain residual cancer cells in the early phase of transplantation (21). Of note, the GVT activity of donor NK cells is significantly improved when KIRs of donor and HLA class I of the recipient are incompatible, and consequently when inhibitory signals are absent, as observed in HLA-haploidentical HSCT (22). Therefore, increased GVT activity of NK cells with KIR-HLA incompatibility is the underlying rationale for the development of allogeneic NK cell therapy.

### Allogeneic NK cell-based immunotherapy

Following the discovery of inhibitory KIRs and the understanding that they play a role in preventing NK cell killing of self MHC class I-expressing tumor cells, investigators began to research the possibility of using allogeneic donor NK cells instead of autologous NK cells for cancer therapy. Several groups have infused activated, expanded donor NK cells to patients early after allogeneic HSCT to provide antitumor effects (23). In Table 1, clinical trials with allogeneic NK cells as therapeutics are summarized.

**Table 1:** **Selected clinical trials with expanded allogeneic NK cells**.

Diseases	Status	Phase of trials	Cell product	Combined therapy	Institute	ClinicalTrials.gov Identifier
Hepatocellular carcinoma	Ongoing	Phase 2	*Ex vivo*-expanded NK cells	None	Samsung Medical Center, Korea	NCT02008929
Lymphoma and solid tumors	Completed	Phase 1	*Ex vivo*-expanded NK cells	None	Seoul National University Hospital, Korea	NCT01212341
High-risk solid tumors	Ongoing	Phase 2	*Ex vivo*-expanded NK cells	Haploidentical HSCT, RIC, and IL-2	Samsung Medical Center, Korea	NCT01807468
Non-B lineage hematologic malignancies and solid tumors	Completed	Phase 1	*Ex vivo*-expanded haploidentical NK cells	Chemotherapy and IL-2	St. Jude Children’s Research Hospital, USA	NCT00640796
Hematological malignancies	Ongoing	Phase 1	IL-2-activated NK cells	Haploidentical HSCT and RIC	Institut Paoli-Calmettes, France	NCT01853358
Multiple myeloma	Ongoing	Phase 1/2	*Ex vivo-*expanded haploidentical NK cells	Autologous HSCT and chemotherapy	University Hospital, Basel, Switzerland	NCT01040026
Leukemia and myeloproliferative disease	Ongoing	Phase 1/2	*Ex vivo*-expanded NK cells	Haploidentical HSCT, TBI, and chemotherapy	M.D. Anderson Cancer Center, USA	NCT01904136
ALL	Ongoing	Phase 2	K562-mb15-41BBL and IL-2-stimulated NK cells	Haploidentical HSCT and chemotherapy	National University Health System, Singapore	NCT01974479
AML and ALL	Ongoing	Phase 1/2	*Ex vivo*-expanded NK cells	Haploidentical HSCT	Asan Medical Center, Korea	NCT01795378
Relapsed/refractory pediatric acute leukemia	Ongoing	Phase 2	Activated and expanded NK cells	Haploidentical HSCT and salvage chemotherapy	Hospital Universitario La Paz, Spain	NCT02074657
Myelodysplastic syndrome and leukemia	Completed	Phase 1/2	IL-2-activated NK cells	Haploidentical HSCT, chemotherapy, and IL-2	M.D. Anderson Cancer Center, USA	NCT00402558
Leukemia	Completed	Phase 2	IL-2-activated NK cells	Chemotherapy and IL-2	Masonic Cancer Center, University of Minnesota, USA	NCT00274846
Relapsed/refractory pediatric T cell leukemia and lymphoma	Ongoing	Phase 1/2	Activated and expanded NK cells	Salvage chemotherapy	Hospital Infantil Universitario Niño Jesús, Madrid, Spain	NCT01944982
Leukemia	Ongoing	Phase 1/2	mbIL21-expanded haploidentical NK cells	Chemotherapy	M.D. Anderson Cancer Center, USA	NCT01787474
Acute leukemia and myelodysplastic syndrome	Ongoing	Phase 1	K562-mb15-41BBL and IL-2-stimulated NK cells	Immunosuppressive therapy and IL-2	National University Hospital, Singapore	NCT02123836

*HSCT, hematopoietic stem cell transplantation; RIC, reduced-intensity conditioning; ALL, acute lymphocytic leukemia; AML, acute myeloid leukemia; TBI, total body irradiation*.

Allogeneic NK cells can be delivered either in a setting of HSCT or a non-HSCT setting. HSCT is a curative platform for many patients with hematologic malignancies. For patients lacking an HLA-identical donor and for those with progressive disease, the use of HLA-haploidentical family donors is increasingly considered to be a suitable alternative. Therefore, in most clinical trials using allogeneic NK cells, autologous or haploidentical HSCT are followed by NK cell infusion as therapeutics to protect relapse and delay recurrence. Several groups have explored the use of allogeneic NK cells in treating relapses of hematologic malignancies following HLA-haploidentical HSCT in clinical trials, and GVHD did not develop when allogeneic haploidentical NK cells were used (19, 24). In these studies, tumor responses were observed in some patients and overall rates of relapse were reduced. Notably, infusion of allogeneic NK cells can cause cancer regression even without allogeneic HSCT. The patients received allogeneic NK cells without HSCT following non-myeloablative chemotherapy. The chemotherapy pre-conditioning delayed the rejection of the transferred cells, and in some cases, the allogeneic NK population even expanded before being ultimately rejected (25, 26).

In a non-transplantation setting, Miller and colleagues were the first to establish the safety and efficacy of adoptive cellular transfer of HLA-haploidentical NK cells in patients with advanced cancer (27). In this study, 19 acute myeloid leukemia (AML) patients were given haploidentical NK cell infusions together with IL-2 and 5 patients achieved complete remission. Allogeneic NK cells with KIR-HLA mismatches between patients and donors exhibited greater tumor-killing activity without causing GVHD. Based on the success observed in AML, a number of clinical trials are being carried out to determine the feasibility and efficacy of allogeneic NK cell infusion for cancer treatment. Many of 15 ongoing clinical trials are oriented to hematological malignancies including leukemia, multiple myeloma, and myelodysplastic/proliferative diseases. Additionally, clinical trials have shown that allogeneic NK cells play a therapeutic role in solid tumors (26, 28, 29). The clinical efficacy of expanded allogeneic NK cells was investigated in patients with recurrent metastatic breast and ovarian cancers in combination with a Hi-Cy/Flu preparative chemotherapy regimen (29). Adoptive transfer of *ex vivo-*expanded allogeneic NK cells was safe and effective in patients with advanced non-small cell lung cancer (26). These findings provided proof of concept that allogeneic NK cells could be effective not only in hematologic malignancy patients but also in solid tumor patients. Clinical trials are currently carried out in hepatocellular carcinoma and neuroblastoma (NCT02008929, NCT01807468).

Adoptive transfer of allogeneic NK cells that come from a totally unrelated donor has also been demonstrated to be safe without any significant side effects (NCT01212341). Allogeneic NK cell therapy is currently applied to patients with advanced hepatocellular carcinoma after curative resection (NCT02008929). In this clinical trial, *ex vivo*-expanded allogeneic NK cells were administered without combination with other therapeutic modalities to investigate the isolated effect of infused allogeneic NK cells.

Taken together, the clinical studies mentioned above demonstrated that the infusion of allogeneic NK cells after *ex vivo* expansion is largely safe and some responses appear encouraging.

## Optimized Selection of Donors

### Lessons from allogeneic HSCT

In T cell-depleted HSCT, donor NK cells are the major effector cells responsible for controlling residual cancer cells (19), and it has been shown that the KIR genotype of donors influences the outcome of HSCT (30). From the experience of allogeneic HSCT, we can learn how allogeneic NK cell donors are selected to maximize the antitumor activity of infused allogeneic NK cells.

There are two distinct types of KIR haplotypes: group A and group B. The KIR group B haplotype has more activating receptors than the KIR group A haplotype (31). According to the KIR genotype, all individuals can be divided into the A/A genotype (homozygous for A haplotypes) or the B/x genotype (having 1 or 2 B haplotypes). There have been reports that the donor KIR genotype influences outcomes of unrelated HSCT for acute hematological malignancies and that the B/x genotype confers significant survival benefit to patients (22, 32, 33). B/x donors are further differentiated on whether their B haplotype genes are in the centromeric or/and telomeric part. On the basis of this information, the KIR B-content score can be calculated from 0 to 4 (30, 34). High donor KIR B-content scores have been associated with a significantly reduced relapse in children after haploidentical HSCT for acute lymphocytic leukemia (ALL) (35), and donors with two or more B-content scores showed superior survival after unrelated HSCT for AML (27).

Incompatibility between KIRs of donors and HLAs of recipients is also an important factor. Considering that each KIR binds to specific HLA allotypes as an inhibitory ligand (e.g., KIR2DL1 to group 2 HLA-C, KIR2DL2/3 to group 1 HLA-C, and KIR3DL1 to HLA-Bw4), a recipient may lack specific HLA allotypes that inhibit donor NK cells. In this case, higher antitumor activity of donor NK cells is expected. Indeed, antitumor activity of donor NK cells is significantly improved when KIRs and HLAs are incompatible between donor and recipient (19, 24, 36).

In addition to the KIR genotype and incompatibility, actual expression of KIRs on NK cells needs to be considered for the best antitumor activity of allogeneic NK cells because the expression of KIRs occurs in stochastic combination (37). Antitumor activity is likely to be mediated by single-KIR^+^ allogeneic NK cells not encountering any inhibitory signal from HLA molecules on recipient cells (38). Although NK cells are the first lymphoid population to recover after allogeneic HSCT (21), reconstitution of mature NK receptor repertoires requires at least 3 months (39). Importantly, during this period, donor-derived single-KIR^+^ NK cells are not fully functional (38). In this aspect, infusion of single-KIR^+^ mature NK cells selected for KIR-HLA mismatches might lead to better clinical outcomes. Currently, multicolor flow cytometry enables the examination of KIR expression in the NK cell population. The approach to generate GMP-grade single-KIR^+^ NK cells (40) will allow customized allogeneic NK cell therapy.

### Sources of allogeneic NK cells

To permit therapeutic use of allogeneic NK cells in clinical settings, a sufficient number of highly enriched NK cells must be obtained. The sources for allogeneic NK cells include peripheral blood mononuclear cells (PBMCs) collected by leukapheresis from healthy donors and umbilical cord blood (UCB).

Peripheral blood mononuclear cells collected by leukapheresis are generally utilized as a source of allogeneic NK cells. Various methods to obtain *ex vivo*-expanded, activated, and CD3^+^ T cell-depleted NK cells have been well established in clinical scales and grades (41). Although those NK cells showed potent antitumor efficacy *in vitro* and *in vivo*, clinical outcomes were insufficient. The clinical results might be influenced by several factors including malignancy types and pre-conditioning treatment. As described above, the therapeutic efficacy of allogeneic NK cell therapy can be potentiated by optimal selection of NK cell donors in a non-HSCT setting. Since NK cells from haploidentical donors had been used in allogeneic HSCT settings, allogeneic NK cells were mostly obtained from haploidentical donors even in a non-HSCT setting. Recently, *ex vivo*-expanded, allogeneic NK cells from unrelated, random donors were successfully administered to patients with malignant lymphoma or advanced solid tumors in a phase 1 trial (NCT01212341) that has proceeded to a phase 2 trial of patients with hepatocellular carcinoma (NCT02008929). This strategy, which used unrelated NK donors, allowed free selection of the best donor in terms of donor KIR-recipient HLA incompatibility without limitation of small pools of related donors. Furthermore, the use of allogeneic NK cells from non-HLA-related healthy donors allows preparation of expanded, cryopreserved NK cells for instant administration without delay for *ex vivo* expansion.

Umbilical cord blood is another promising source of allogeneic NK cells. However, cytokine-based differentiation of CD34^+^ hematopoietic stem and progenitor cells to NK cells needs to be carried out to obtain large numbers of functional NK cells from UCB (42). This process requires high-dose cytokine cocktails and delicate culture regimens that may result in low-cost effectiveness. Recently, an NK cell expansion method from UCB using artificial antigen presenting feeder cells was reported. NK cells expanded by this method showed *in vitro* cytotoxicity against various myeloma targets and *in vivo* antitumor activity in a mouse model of myeloma (43).

## Future Directions

### Genetic modification

Genetic modification is a promising option for redirecting the function of various types of immune cells (44). Much work has been performed, particularly on genetically redirecting T cells against a range of tumor antigens. For example, T cells expressing chimeric antigen receptors (CARs) targeting CD19 antigens have been developed to treat B-cell-derived malignancy, and clinical trials are currently ongoing (45–47). The successful experience with CAR-expressing T cells in the treatment of hematological malignancies has prompted the development of CAR-expressing NK cells. NK cells are attractive for CAR expression because they have cytotoxic function and, unlike T cells, allogeneic NK cells do not cause GVHD.

As summarized in Table 2, two clinical trials are investigating the use of CAR-expressing allogeneic NK cells. The aim of both studies is to assess the safety, feasibility, and efficacy of expanded, activated, and CD19-redirected haploidentical NK cells in ALL patients who have persistent disease after intensive chemotherapy or HSCT (NCT00995137, NCT01974479). Further, other tumor antigens, such as CS1, CEA, CD138, and CD33, are targeted by CARs expressed by NK cells, although NK-92, YT, or NKL cell lines were used (48–51).

**Table 2:** **Genetically modified, expanded allogeneic NK cells**.

Modification	Genes transferred	NK cells	Application	Status	Reference/ClinicalTrials.gov Identifier
Target specificity	CD19	Haploidentical, expanded NK cells	ALL	Phase 1	St. Jude Children’s Research Hospital (NCT00995137)
	CD19	Haploidentical, expanded NK cells	ALL	Phase 2	National University Health System, Singapore (NCT01974479)
	CD19	Expanded NK cells	B-ALL	Preclinical	Cho *et al*. (52)
	CD20	Expanded NK cells	CD20^+^ B-NHL	Preclinical	Chu *et al*. (53)
	GD2	Expanded NK cells	Neuroblastoma	Preclinical	Esser *et al*. (54)
NK cell function	NKG2D	Expanded NK cells	Various tumor targets (B-ALL etc.)	Preclinical	Chang *et al*. (55)
	IL-12	IL-2-activated NK cells	B16 lung tumor	Preclinical	Goding *et al*. (56)

*ALL, acute lymphocytic leukemia; B-NHL, B-cell non-Hodgkin lymphoma; B-ALL, B-cell acute lymphoblastic leukemia*.

Genetic modification is also performed to express cytokine transgenes in NK cells. NK cell function could be enhanced by expression of cytokines, such as IL-2 (57, 58), IL-12 (56, 59), and IL-15 (60–62). Cytokine expression enhances the activation of NK cells, survival and proliferation of NK cells, and accumulation of NK cells in tumor tissues. To improve the efficacy of NK cell therapy, genetic modification of NK cells is explored to express activating receptors, such as NKG2D (55).

### Therapeutic regimens

In allogeneic NK cell therapy, optimal therapeutic regimens for clinical applications should be considered because adoptively transferred NK cells not only target tumor cells but also interact with the immunological environment. To potentiate the therapeutic efficacy of allogeneic NK cells, proper strategies, including pre-conditioning or combination therapy, could be applied (34).

Upregulation of NKG2D ligands by spironolactone (63) or histone deacetylase inhibitors (64, 65) and upregulation of TRAIL-R2 by doxorubicin (66) result in enhanced antitumor efficacy of NK cells. Proteasome inhibitors also sensitize tumor cells to NK cell-mediated killing via TRAIL and FasL pathways. In addition, c-kit tyrosine kinase inhibitor (67) and JAK inhibitors (68) increase the susceptibility of tumor cells to NK cytotoxicity and enhance antitumor responses by increased IFN-γ production from NK cells. However, protein kinase inhibitors should be used cautiously because some protein kinase inhibitors, such as sorafenib, inhibit the effector function of NK cells (69).

Immunomodulatory drugs can augment NK cell function. Lenalidomide enhances rituximab-induced killing of non-Hodgkin’s lymphoma and B-cell chronic lymphocytic leukemia through NK cell and monocyte-mediated ADCC mechanisms (70). Combination therapy using IL-2 and anti-CD25 shows anti-leukemic effects by depletion of regulatory T cells in addition to activation and expansion of NK cells (71). Alloferon, an immunomodulatory peptide, enhances the expression of NK-activating receptor 2B4 and granule exocytosis from NK cells against cancer cells (72).

Therapeutic antibodies can be combined with allogeneic NK cell therapy (73). Antibodies against tumor antigens (e.g., CD20 and CS1) can induce ADCC of NK cells (74, 75). Antibodies to activating NK receptors (e.g., 4-1BB, GITR, NKG2D, DNAM-1, and NCRs) can enhance NK activation (74, 76–79). In addition, inhibitory receptors (e.g., KIR2DL, PD-1, PD-L1, and NKG2A) can be blocked by antibodies (80–85). Bispecific and trispecific killer cell engagers directly activate NK cells through CD16 signaling and thus, induce cytotoxicity and cytokine production against tumor targets (86, 87).

## Conclusion

Antitumor activity of allogeneic NK cells was first observed in a setting of HLA-haploidentical HSCT. Allogeneic NK cell therapy was tried mostly using HLA-haploidentical NK cells with or without allogeneic HSCT and, recently, allogeneic NK cells from unrelated, random donors have been used in a non-HSCT setting. The efficacy of allogeneic NK cell therapy can be enhanced by optimal donor selection in terms of the KIR genotype of donors and donor KIR-recipient MHC incompatibility. Furthermore, efficacy can be increased by genetic modification of NK cells and optimized therapeutic regimens. In the future, allogeneic NK cell therapy can be an effective therapeutic modality for cancer treatment.

## Conflict of Interest Statement

Yu Kyeong Hwang is a current employee of GreenCross LabCell. The other co-authors declare that the research was conducted in the absence of any commercial or financial relationships that could be construed as a potential conflict of interest.
